# Improving estimates of loss-of-function constraint for short genes

**DOI:** 10.1038/s41588-024-01829-0

**Published:** 2024-08-01

**Authors:** Nicola Whiffin

**Affiliations:** Big Data Institute and Centre for Human Genetics, University of Oxford, Oxford, UK

## Abstract

Genetic constraint identifies genes under selection against loss-of-function, but existing methods are inaccurate for shorter genes. A new study overcomes this key limitation to ascribe more confident predictions to all human protein-coding genes.

Genetic constraint is a term used to collectively describe techniques that assess the strength of negative selection acting on genetic variation^1^. Constraint is calculated for a group of variants, most frequently of the same functional class (for example, predicted loss-of-function (pLOF) variants) and/or across specific regions (for example, protein-coding regions of genes). If a genomic region is ‘intolerant’ to a certain class of variation, then natural selection will purge variants in the region of that class from the population. The strength of this effect can be estimated using statistical models.

There are two broad groups of approaches used to model genetic constraint: (1) methods that assess the allele frequency of variants in a population, with variants under selection appearing at lower frequencies than those that are evolving neutrally, and (2) methods that measure a depletion in the number of unique variants that are observed in a population compared to an expected number. The statistical models underlying constraint metrics vary greatly in their complexity, with recent models incorporating numerous features that are known to influence ‘mutability’ (that is, the absolute chance of any variant occurring) in an attempt to increase model accuracy^2^.

The majority of constraint metrics that have been developed to date assess selection acting on pLOF variants (that is, those that are predicted to render a single copy of the gene non-functional) at the level of individual genes. Examples include pLI^3^, LOEUF^4^, and *s*_het_^5^. These metrics enable identification of genes within which heterozygous pLOF variants are probably deleterious. This knowledge has wide utility, including aiding disease-gene discovery^6–8^, improving interpretation of the effect of individual variants in disease^9^ and supporting the evaluation of potential drug targets^10,11^.

A key limitation in measuring gene-level constraint against pLOF variants is that these variants are inherently rare. This is a particular issue for shorter genes with few observed and expected pLOF variants. Simply put, prior constraint models do not have enough information to accurately estimate constraint for the shortest roughly 25% of genes. As a result, current methods take a conservative approach and classify the majority of short genes as unconstrained. Take for example *DCX*, which encodes a 366-amino-acid-long protein and has only three observed pLOF variants in the Genome Aggregation Database (gnomAD; the dataset used to calculate both LOEUF and *s*_het_). There are 56 pathogenic pLOF variants in *DCX* in ClinVar, with these variants reported to cause dominant X-linked lissencephaly, a severe brain malformation disorder with a short life expectancy^12^. Despite high levels of negative selection expected to act on pLOF variants in *DCX*, it is not classified as constrained by LOEUF or pLI.

A recent paper from Zeng et al.^13^ reports an updated pLOF constraint model that aims to combat this limitation and ascribe more confident *s*_het_ predictions to shorter genes (Fig. 1). The authors use a machine-learning-based approach to learn gene-level features that are predictive of constraint from genes with confident *s*_het_ estimates. The final model includes 1,248 features including patterns of gene expression across tissues, biological pathway and protein network information, the number and length of regulatory elements, cross species conservation, gene structure information, constraint against missense variants, and gene ontology terms. Models built using these predictive features are then used to make more confident predictions for those problematic short genes for which there is not enough information to estimate *s*_het_ from observed variants alone. As expected, the updated *s*_het_ model is highly correlated with LOEUF for longer genes but the two metrics have much lower correlation for genes with few expected pLOF variants. The authors note many examples of known disease genes that were unconstrained by LOEUF but that are now classified as constrained by *s*_het_. One of those examples is *DCX*. Reassuringly, the gene features that are most predictive of constrained genes include expression patterns and gene ontology terms for brain and development, consistent with strong selection acting on early-onset conditions and severe developmental disorders.

Another confounder in constraint measurements is the high likelihood that pLOF variants that we do observe in the population are either sequencing or annotation errors^14,15^. The latter being variants that although initially annotated as pLOF do not render the allele non-functional, normally due to some form of ‘rescue’. For example, a variant affecting a splice acceptor site can be rescued by the existence of a nearby alternative acceptor site that is in the same frame. The confounding effect of these mis-annotation errors is most pronounced for genes under the strongest levels of negative selection, where true null loss-of-function variants are embryonic lethal; in these genes all observed pLOF variants must be sequencing or annotation errors, or have arisen somatically.

The earliest pLOF models, including pLI, estimated constraint across all annotated pLOFs, without accounting for sequencing errors. LOEUF uses the loss-of-function transcript effect estimator (LOFTEE) tool^4^ to remove pLOF variants matching common reasons for annotation errors (for example, those in the final exon that do not cause transcript degradation through nonsense-mediated decay) before estimating constraint. In their new *s*_het_ model, Zeng et al.^13^ instead used an elegant statistical approach to explicitly model the probability that each pLOF variant is mis-annotated. To do this, they use information on both the number of unique pLOF variants observed in the gene and their allele frequencies. This is in contrast to previous approaches that use either counts of unique pLOF variants or their aggregated allele frequencies, often ignoring valuable information.

Although much attention has been given to refining models of pLOF constraint, such as in this study, modelling genetic constraint has considerable utility outside of the context of pLOF variants and gene-level measures. Indeed, Zeng et al.^13^ note in their discussion that extending their approach to non-coding variation would be an interesting future direction. Previous non-coding constraint models have suffered with a lack of resolution and difficulty in calibrating mutational models across the whole genome, particularly at the start of genes^2^. Solving these issues will be difficult, but a genome-wide constraint map at the resolution of individual regulatory elements would be invaluable for decoding the entire genome.

## Figures and Tables

**Fig. 1:**
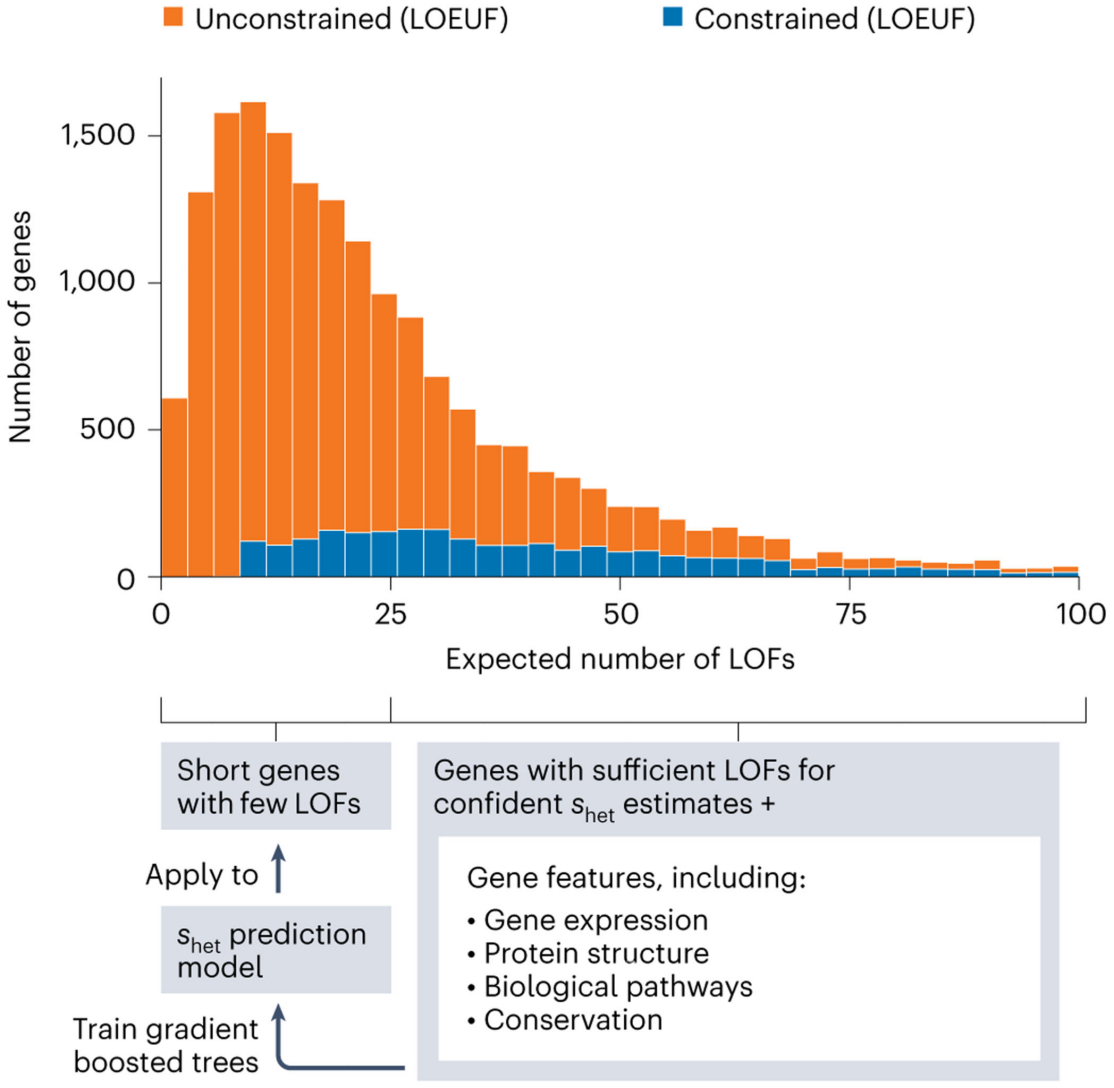
A machine-learning model incorporating gene features improves genetic constraint estimates for short genes. Top, stacked histogram of the expected number of LOF variants per gene colored by LOEUF constraint scores (unconstrained: LOEUF < 0.35; constrained: all other genes). Bottom, schematic representation of the approach used by Zeng et al.^13^ to improve estimates of *s*_het_ for shorter genes. Adapted from ref. 13, Springer Nature Limited.

## References

[1] Fuller ZL, Berg JJ, Mostafavi H, Sella G, Przeworski M (2019). Nat Genet.

[2] Chen S (2024). Nature.

[3] Lek M (2016). Nature.

[4] Karczewski KJ (2020). Nature.

[5] Cassa CA (2017). Nat Genet.

[6] Seaby EG (2022). Genet Med.

[7] He X (2013). PLoS Genet.

[8] Kaplanis J (2020). Nature.

[9] Gudmundsson S (2022). Hum Mutat.

[10] Minikel EV (2020). Nature.

[11] Whiffin N (2020). Nat Med.

[12] Pilz DT (1998). Hum Mol Genet.

[13] Zeng T (2024). Nat Genet.

[14] Singer-Berk M (2023). Am J Hum Genet.

[15] MacArthur DG, Tyler-Smith C (2010). Hum Mol Genet.

